# Identification of Protein Network Alterations upon Retinal Ischemia-Reperfusion Injury by Quantitative Proteomics Using a *Rattus norvegicus* Model

**DOI:** 10.1371/journal.pone.0116453

**Published:** 2014-12-30

**Authors:** Han Tian, Leilei Wang, Ruiqi Cai, Ling Zheng, Lin Guo

**Affiliations:** College of Life Sciences, Wuhan University, Wuhan, China; University of Cincinnati, College of Medicine, United States of America

## Abstract

Retinal ischemia is a common feature associated with several ocular diseases, including diabetic retinopathy. In this study, we investigated the effect of a retinal ischemia and reperfusion (I/R) injury on protein levels via a quantitative shotgun strategy using stable isotope dimethyl labeling combined with LC-MS/MS analysis. Based on the relative quantitation data of 1088 proteins, 234 proteins showed a greater than 1.5-fold change following I/R injury, 194 of which were up-regulated and 40 were down-regulated. Gene ontology analysis revealed that after I/R injury, there was an increase in the metabolic-process related proteins but a decline in cell communication, system process and transport-related proteins. A ribosome protein network and a secreted protein network consisting of many protease inhibitors were identified among the up-regulated proteins, despite a suppression of the mammalian target of rapamycin (mTOR) pathway following the I/R injury. A synaptic-related protein network was found to be significantly down-regulated, implicating a functional reduction of neurons following a retinal I/R injury. Our results provide new systems-biology clues for the study of retinal ischemia.

## Introduction

Diabetic retinopathy, one of the most common complications in diabetic patients, can eventually lead to blindness [1]. The large and ever-increasing number of diabetics worldwide implies that diabetic retinopathy will continue to be a major cause of blindness and visual impairment in the foreseeable future. Diabetic retinopathy, and most ocular morbidity, is either directly or indirectly a result of retinal ischemia. Rodent models of retinal ischemia induced by high intraocular pressure followed by reperfusion (I/R) lead to neuronal and vascular degeneration, which mimics the morphological changes in several retinal diseases, including diabetic retinopathy. Thus, this model has been widely used to investigate the molecular mechanisms of the pathogenesis of retinal diseases and to identify potential therapeutic strategies to prevent them [2].

Several cellular processes have been linked to retinal degeneration after I/R injury, including glutamate excitotoxicity [3]–[5], oxidative stress [6]–[8], and inflammation [9]–[12]. Previous studies have primarily focused on specific processes or pathways; however, the pathogenesis of retinal degeneration after I/R injury is likely the result of interactions between several pathways.

In recent years, the proteomics approach has become a widely used strategy for large-scale screening to elucidate changes at the protein level [13]. Several studies have used this approach to identify changes in the signaling pathways in rodent models of retinal diseases. By using a two-dimensional differential gel electrophoresis (2D-DIGE)-based approach, Zheng et al. identified a total of 96 protein spots that were significantly altered following an I/R injury [14]. A number of the altered proteins were linked to glycolysis and carbohydrate metabolism, and some of the protein level changes could be normalized to physiological levels by pharmacological treatment. In a mass spectrometry (MS)-based label-free quantitative proteomics study, a total of 328 proteins were quantified and reported in a rat model [15]. In a recent study, we applied a label-free proteomics strategy to quantify changes in the histone post-translational modifications (PTMs) upon I/R injury and found 34 significant histone PTM changes (p<0.05), providing a detailed profile for histone-based epigenetic regulation of the I/R injury response [16]. Despite the recent progress, the proteome coverage in the above mentioned global proteomics studies are somewhat limited [14], [15]. This is primarily due to the intrinsic limitations of the 2D-based proteomics strategy and technical challenges in the label-free mass spectrometry quantitation approach.

To gain further insights into the potential mechanisms that contribute to retinal degeneration in the I/R model, a stable isotope-based quantitative proteomics method was used in this study. Stable isotope dimethyl labeling of peptides is a relatively simple and low-cost quantitative proteomics method based on chemical labeling [17]. When used as an alternate metabolic labeling method, dimethyl labeling can be applied to both cell and tissue protein samples and is commonly used for tissue samples where SILAC (stable isotope labeling by amino acids in cell culture) labeling is not feasible. Using this method, we quantified 1088 proteins and found 234 that were altered more than 50% (threshold of 1.5-fold) in the retina following an I/R-injury. Among the altered proteins, proteins involved in the ‘metabolic process’ category were enriched in the up-regulated proteins, while those involved in ‘cell communication’, ‘system process’ and ‘transport’ were overrepresented in the down-regulated proteins following the injury. Furthermore, using a STRING analysis [18], a noticeable up-regulation of ribosomal proteins and a down-regulation of synapse-related proteins were found. Through western blot and immunohistochemistry (IHC) studies, we found that the mTOR pathway was suppressed in the retina in despite of the increase in ribosomal proteins, and the synaptic proteins were down-regulated significantly following the I/R injury. Our results provide new insights to elucidate the mechanism of retinal degeneration in the I/R-injury model.

## Materials and Methods

### Ethics Statement

All of the procedures involving the animals conformed to the Association for Research in Vision and Ophthalmology Statement for the Use of Animals in Ophthalmic and Vision Research and was approved by the Committee on Ethics in the Care and Use of Laboratory Animals of Wuhan University.

### Rat Model of Retinal I/R Injury

Male Wistar rats (160–200 g) were obtained from the Hubei Animal Laboratory (Wuhan, China) and housed in ventilated microisolator cages with free access to water and food. The retinal I/R injury was induced as previously described [19], [20]. The duration of ischemia was 60 min. Following the ischemia, the reflow of the retinal circulation was documented visually. For each rat, the left retina was used for the I/R injury, and the right retina served as the control. The animals were sacrificed 2 or 5 days after the I/R injury. To isolate the retina samples, corneal was cut off and lens was drug out, then retinal sample was obtained.

### Cell Culture and Ischemia and Reperfusion Treatment *in Vitro*

Human neuroblastoma cells lines (SH-SY5Y) was a gift from Dr. Zan Huang (College of life sciences, Wuhan University). Cells were cultured in DMEM media (Hyclone South Logan, UT) supplemented with 10% FBS (Gibco, Grand Island, NY) and 1% penicillin-streptomycin (Hyclone, South Logan, UT) (named as cell growth media) at normal culture condition.

After the cell reached full confluence in the plate, *in vitro* ischemia and reperfusion was conducted. The culture media was replaced by DMEM medium (Gibco, Grand Island, NY) without glucose and FBS, and then settled immediately in hypoxic chamber (Thermo scientific, Marietta, OH) with the condition of 1% O_2_, 5% CO_2_ at 37°C, which is regard as ischemia [21]. After 2 hours, the cells were re-cultured in cell growth media. And another 12 hours (reperfusion) later, the cells were collected.

### Protein Sample Preparation

Freshly isolated retinas were homogenized in a hypotonic lysis buffer containing PhosSTOP (Roche, Basel, Switzerland) and cOmplete protease inhibitor cocktail (Roche). The retinal homogenate and the collected cells were further lysed with RIPA (Beyotime, China) hypotonic lysis buffer as previously described [20]. The protein concentrations were measured, and the samples were stored at −80°C.

Equal amounts of protein from 4 retinas (100 µg from each) with the same treatment were mixed together into two groups: the control retinas and the I/R-treated retinas. As a biological replicate, an additional group of four rats was treated and all of the above experiments were repeated. The proteins from the different groups were reduced with 10 mM DTT, alkylated with 40 mM iodoacetamide and digested separately with trypsin (1∶50, trypsin:protein) as previously described [22].

For the stable isotope dimethyl labeling, the digested peptides from the control and I/R-treated retinas were reconstituted separately with 200 µl of CH_3_COONa (pH 5.9). Forty microliters of CH_2_O (light labeled) and 40 µl of CD_2_O (heavy labeled) were added to the control and I/R-treated retinal samples, respectively. Forty microliters of 0.6 M NaBH_3_CN was added to each sample and incubated at room temperature for 30 min. To quench the reaction, 160 µl of a 1% (v/v) ammonia solution and 80 µl of 5% formic acid were added to the samples on ice. The light-labeled and heavy-labeled samples were mixed and desalted with 50-mg Sep-Pak C18 Cartridges (Waters, Milford, MA).

### Strong Cation Exchange (SCX) Fractionation and LC-MS/MS Analysis

For the strong cation exchange (SCX) fractionation, the mixed peptides were resuspended in buffer A containing 5 mM KH_2_PO_4_ and 20% acetonitrile, pH 2.7. The SCX was performed on a polysulfoethyl column (2.1×50 mm, 5 µm×200 Å) and used a KCl gradient from 0 to 0.5 M in 50 min to fractionate the peptides. Twelve fractions were collected and desalted using C18 ZipTips (Millipore, Billerica, MA) before the MS analysis.

A QSTAR ELITE mass spectrometer (Applied Biosystems, Foster City, CA) coupled with a nanoflow HPLC system (Tempo™, Applied Biosystems) was used for the relative quantitation of the retinal proteome before and after the I/R treatment. The LC-MS/MS method was performed as previously described [23]. Briefly, a 130-min liquid chromatography (LC) gradient was used to separate the peptide mixture with mobile phase A (2% ACN, 0.1% formic acid) and mobile phase B (98% ACN, 0.1% formic acid). The IDA (information dependent acquisition) mode was used to generate the MS/MS data. For the MS scan, the m/z range was set from 400 to 1800 with a charge state from 2 to 5, and each MS scan was followed by 5 MS/MS events. The raw MS data were generated by Analyst QS 2.0 (Applied Biosystems).

### Data Analysis

The raw data from the QSTAR ELITE were analyzed with MASCOT Daemon software (version 2.2.2, Matrix Science) using a local Mascot server. The data were searched against the SwissProt Rattus database with added dimethyl masses. The following parameters were set for the Mascot searches: Cysteine carbamidomethylation was selected as a fixed modification, whereas methionine oxidation and serine, threonine and tyrosine phosphorylation were selected as variable modifications. A maximum of two missed cleavages were allowed. The precursor and fragment ion tolerance were set as 100 ppm and 0.4 Da. The peptide charge was set to 2+ and 3+ and the significance threshold was set at p<0.05. One unique peptide was used to identify a protein. The false-positive rates of the two biological replicates were 1.16% and 1.14%, for the first and second replicates, respectively.

Following the Mascot search, the generated rov files were opened using Mascot Distiller (version 2.3.2.0, Matrix Science) for quantitation. For the quantitation, the three parameters, ‘fraction’, ‘correlation’ and ‘standard error’, were set at 0.5, 0.9 and 0.2, respectively. The weighted average of all of the peptides was used to normalize the peptide ratios, and the protein ratios were calculated as the weighted average ratios with the normalized peptide ratios.

### Bioinformatics Analysis

Proteins with at least a 1.5-fold change in two replicates were considered to be significantly up-regulated or down-regulated (considered to be ‘altered proteins’). The proteins were classified into biological processes using the PANTHER 8.1 classification system [24]. The total proteins and altered proteins were classified separately. To evaluate whether the categories from the altered proteins were statistically overrepresented when compared to the overall quantitative proteome, the p-values were calculated using a hypergeometric probability distribution that was implemented in the R-language described in Krishnan’s paper [25]. The hypergeometric probability distribution describes the expectation of finding n proteins within a specific category in an amount of m changed proteins, given that there were N proteins with the same category in an amount of M total quantified proteins. For each category, by comparing the actual amount of n proteins with the expected amount of n proteins, the p-values were calculated and a p-value<0.05 was considered to be statistically enriched. All of the changed proteins were submitted to STRING 9.1 for protein interaction network analyses [18]. The results were further processed using Cytoscape (version 2.6.3) [26], an open source software platform for visualizing complex networks and integrating them with any available attribute data.

### Western Blot Analysis

Five microgram of the proteins from *in vivo* and *in vitro* samples were fractionated by SDS-PAGE, electroblotted onto a PVDF membrane (Millipore, Billerica, MA) and probed with different antibodies: anti-Calretinin (1∶5000 dilution, #2449-1, Abcam, CA), anti-actin (1∶2000 dilution, #CW0096B, CWBIO, China), anti-Albumin (1∶10000 dilution, 16475-1-AP, Proteintech, China), anti-synaptophysin (SYPH) (1∶50000 dilution, #1485-1, Abcam, CA), anti-SYT1 (1∶10000 dilution, 14511-1-AP, Proteintech, China), anti-mTOR (1∶1000 dilution, #2983, CST, MA), anti-phospho-mTOR (Ser2448) (1∶1000 dilution, #2971, CST, MA), anti-phospho-p70 S6 Kinase (Ser371) (1∶1000 dilution, #9208, CST, MA), anti-4E-BP1 (1∶1000 dilution, #9644, CST, MA), anti-phospho-4E-BP1 (Thr37/46) (1∶1000 dilution, #2855, CST, MA), anti-GFAP (1∶5000 dilution, #7260, Abcam, CA), anti-vimentin (1∶10000 dilution, #92560, Millipore, MA), anti-ApoA4 (1∶3000 dilution, #5700, CST, MA), anti-GNAL (1∶2000 dilution, #DF4108, Affinity, OH), anti-HSP90AB1 (1∶1000 dilution, #A1087, ABclonal, MA), anti-H2B (1∶1000 dilution, #8135, CST, MA), anti-Annexin A1 (1∶2000 dilution, 21990-1-AP, Proteintech, China) and anti-calnexin (1∶5000 dilution, #2692-1, Abcam, CA). The protein bands were visualized with Immobilion Western HRP and AP Chemiluminescent Substrates (Millipore, Billerica, MA) and evaluated by the Quantity One 1-D Analysis Software (Bio-Rad, Hercules, CA). The expression levels of the target proteins were first quantitated relative to the presence of beta-actin in the same sample, and then the relative protein expression levels in the different groups were normalized to the control groups, with the control amount set as 1-fold. All of the results were expressed as the mean ± SD. The data were analyzed by the Mann-Whitney test. Differences were considered to be statistically significant when the p-values were less than 0.05.

### Morphological Examination and Immunofluorescence Staining

The enucleated eyes were routinely fixed in 10% buffered formalin and embedded in paraffin. The paraffin-embedded sections were deparaffinized in xylene and rehydrated in decreasing concentrations of ethanol. One set of sections was stained with H &E for light microscopy examination. The remaining sets were used for immunofluorescence studies. The sections were first blocked with 2% BSA in PBST, and the primary antibodies, anti-SYPH (1∶1000 dilution, 17785-1-AP, Proteintech, China) and anti-SYT1 (1∶50 dilution, 14511-1-AP, Proteintech) were applied on the sections at 4°C overnight. After washing three times with PBST, the slides were then incubated with the secondary antibody (1∶200 dilution, #R0156, Dako, Denmark) for 1 h. The sections were covered with DAPI and anti-fading medium after extensive washing with PBS and imaged via an Olympus FV1000 Confocal Laser Scanning Microscope (Olympus, Japan).

## Results

### MS Analysis of the Retinal Proteome after I/R Injury

**Figure 1:**
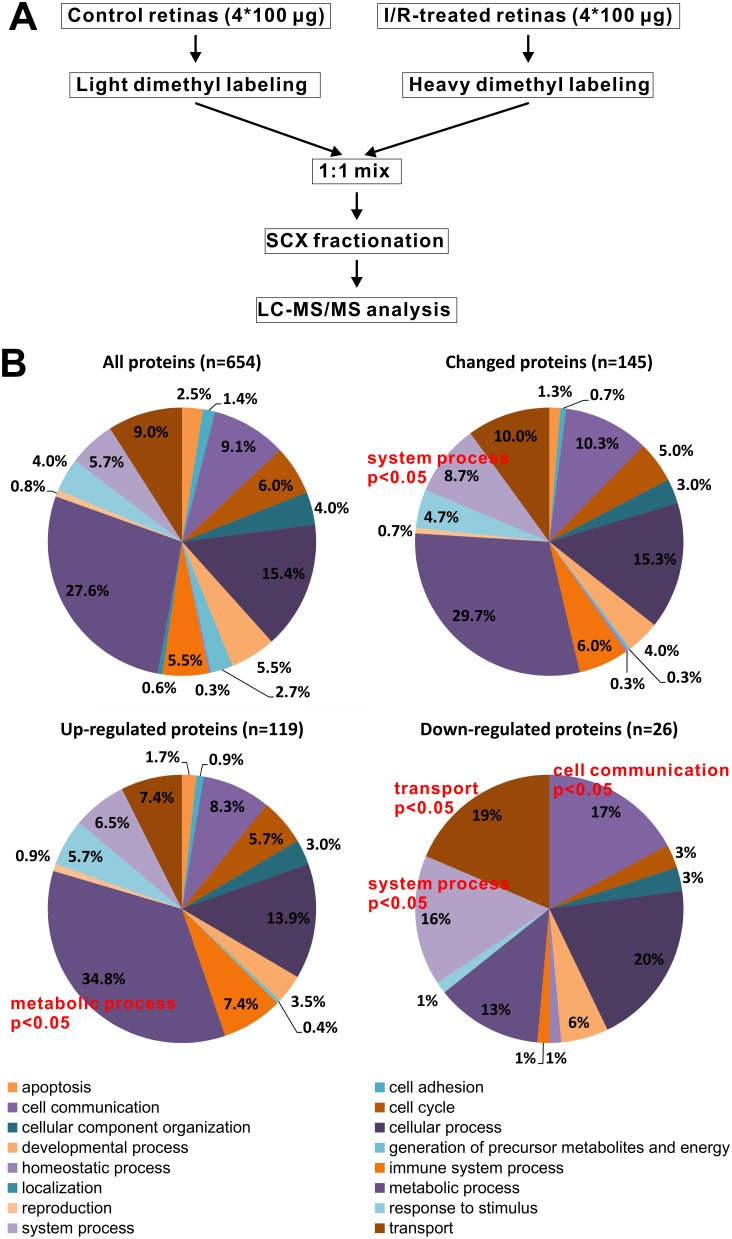
Experimental strategy and data overview. A, Workflow of the dimethyl labeling-based quantitative proteomics analysis, comparing the retinal proteome before and after the I/R treatment; B, The Gene Ontology analysis of biological process. The GO analysis was performed with the PANTHER 8.1 classification system. Upper left, GO classification of the total quantitative proteins as a background; Upper right, GO classification of all of the altered proteins; Down, GO classification of the up-regulated and down-regulated proteins following the retinal I/R injury. P<0.05 represents an enriched category.

To examine the effect of the I/R treatments on rats, we analyzed PASH-stained retinal sections by microscope. As shown in S1 Fig., the I/R induced severe neuron loss at 2-days and 5-days post-I/R injury, similar to previous reports [19], [20]. The proteomics strategy used in the present study is outlined in Fig. 1A. The proteins from individual retinas were extracted separately, and equal amounts of proteins from 4 individual rats were combined as a group. The proteins from the I/R-treated and the control group were reduced with DTT, alkylated with iodoacetamide, and digested with trypsin. The peptides from the control group were labeled with CH_2_O (‘light form’), while the I/R-treated group was labeled with CD_2_O (‘heavy form’). The labeled peptide mixture was mixed in a 1∶1 ratio, and the mixed peptides were fractionated by SCX into 12 fractions, followed by LC-MS/MS analysis.

Two biological replicates were performed and analyzed independently. In the first replicate, 1206 proteins were identified and 993 proteins were quantified with a false-positive rate of 1.16%. In the second replicate, 1134 proteins were identified and 885 proteins were quantified with a false-positive rate of 1.14%. When the two biological replicates were combined, a total of 1223 proteins were identified, with 1117 proteins shared by both (S2A Fig.). Meanwhile, a total of 1088 proteins were quantified with 790 proteins shared by both replicates (S2B Fig.) (S1 Table).

Approximately 22% of the proteins quantified (234 proteins out of 1088 proteins) showed a greater than 1.5-fold change, including 194 up-regulated and 40 down-regulated proteins. This indicates that after I/R injury, there is a dramatic change in the retina proteome, suggesting that a complex underlying protein network may be involved in retinal I/R-injury response.

### Biological Processes Involved in the I/R Response

To determine what biological processes were affected following the I/R injury, we performed a Gene Ontology analysis using the PANTHER classification system [24] (Version 8.1). For the 1088 quantitative proteins, 654 genes were classified in the Gene Ontology database. These 654 genes contributed to 1389 hits in the ‘biological process’ category, which covers 16 main categories (Fig. 1B, upper left). Among the 234 proteins with a greater than 1.5-fold change observed, 145 genes were classified in the Gene Ontology database, with 300 hits in ‘biological process’ and belonging to 15 categories (Fig. 1B, upper right). To evaluate whether the categories of the changed proteins were statistically altered when compared to the overall quantitative proteome, we compared the changed profile to the overall profile and analyzed whether any group was overrepresented in the changed proteins. Using a hypergeometric probability distribution, ‘system process’ was found to be overrepresented (p-value <0.05) in the changed protein category (Fig. 1B, upper right). When we split the changed proteins into two parts, up- and down-regulated, and again compared the changed profiles to the entire quantitative proteome set, we found that in the up-regulated proteins, ‘metabolic process’ was overrepresented (p<0.05) (Fig. 1B, bottom left). Meanwhile, in the down-regulated proteins, we identified three biological processes, ‘cell communication’, ‘system process’ and ‘transport’, that were overrepresented (p<0.05) (Fig. 1B, bottom right). These data suggest that following I/R injury, essential metabolic activities that are critical for cell survival may be elevated, but activities related to cellular communication, system process and transport may be suppressed. Based on these observations, we hypothesize that the selective up- and down-regulation of certain biological processes may be part of a coordinated cellular response to I/R injury.

### Network Analysis of the Proteins Involved in I/R Injury

**Figure 2:**
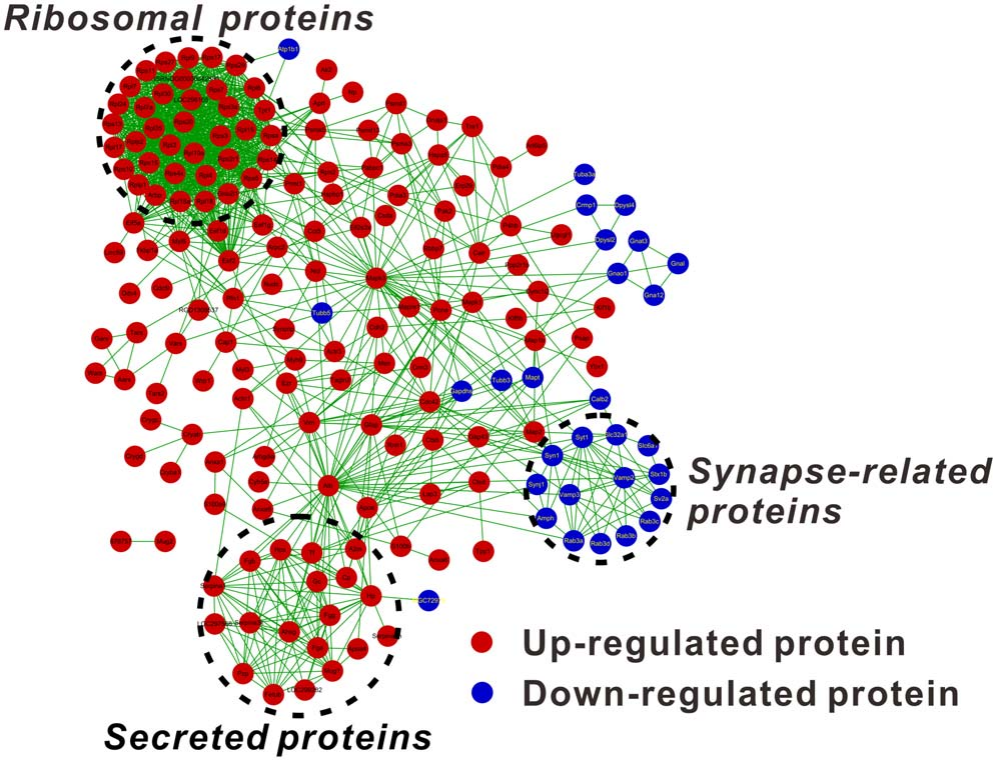
The protein-protein interaction network of the changed proteins reveals a ribosomal, a secreted and a synapse-related protein cluster. The up- or down-regulated proteins were submitted for network analysis using STRING. The obtained proteins and ‘interactions’ were then imported into Cytoscape for data visualization. Only the interactions above a medium confidence level, as defined by STRING (>0.400), are presented.

To analyze whether the I/R-induced proteins have any interactions, the changed proteins were subjected to protein-protein interaction and network analyses using STRING software [18]. Based on the STRING analysis, a complicated protein-protein interaction network was identified (Fig. 2).

For the up-regulated proteins (Fig. 2, shown in red), two groups of proteins clustered together. One group was composed of a ribosome protein network (37 proteins) and the other was a secreted protein network (19 proteins) consisting of primarily protease inhibitors and plasma proteins (Fig. 2). Several other up-regulated proteins were shown to have no clear interaction patterns or functional connections. Albumin is the most abundant protein in plasma However, it appears that Mapk3 and Alb (albumin) are key proteins in the up-regulated protein networks, because these proteins have 40 and 41 edges respectively in the up-regulated protein networks. However, because albumin is the most abundant protein in plasma, we performed IHC analysis of albumin, and found no significant differences between the control and the I/R treated retinas (S3 Fig.). Therefore, the up-regulation of albumin may be caused by contamination during I/R treatment and sample preparation.

For the down-regulated proteins (Fig. 2, shown in blue), one noticeable protein network was identified, consisting of 14 proteins belonging to synapse-related proteins. This indicates that suppression of the synapse and its related biological processes may be critical for retinal neurodegeneration. Two other relatively small protein networks that were identified were a family of dihydropyrimidinase-related proteins (Crmp1, Dpysl2, Dpsyl4) and a family of guanine nucleotide-binding proteins (Gnao1, Gnal, Gna12, Gnat3).

The above protein network analysis categorized only 215 of the altered proteins into protein networks, with 36 proteins remaining unconnected (Table 1). Among the unconnected proteins, seventeen proteins showed a greater than 2.0-fold change, which may be of interest. Among the up-regulated proteins, we identified three retinal structural and matrix proteins: CRGF (Gamma-crystallin F), CRBS (Beta-crystallin S) and IMPG2 (Interphotoreceptor matrix proteoglycan 2). The remaining proteins were primarily metabolic enzymes, such as OAT (Ornithine aminotransferase), and transport-related proteins, such as SC31A (Protein transport protein Sec31A). Among the down-regulated proteins, we identified SYUB (β-synuclein), which is a synaptic protein, and other primarily calcium-binding proteins, including CAMKV (CaM kinase-like vesicle-associated protein), HPCL4 (Hippocalcin-like protein 4) and PITM1 (Membrane-associated phosphatidylinositol transfer protein 1). These unconnected changed proteins currently lack evidence of involvement in the I/R response but may be important for explaining the mechanism of retinal I/R injury.

**Table 1:** Unclassified proteins in the STRING network analysis.

Protein names	Accession	Ratio
**Membrane-associated phosphatidylinositol transfer protein 1**	PITM1_RAT	0.216
**CaM kinase-like vesicle-associated protein**	CAMKV_RAT	0.248
**Beta-synuclein**	SYUB_RAT	0.458
**Hippocalcin-like protein 4**	HPCL4_RAT	0.474
**FXYD domain-containing ion transport regulator 6**	FXYD6_RAT	0.504
**Solute carrier family 12 member 5**	S12A5_RAT	0.515
**Heat shock protein beta-6**	HSPB6_RAT	0.559
**Calcium-dependent secretion activator 1**	CAPS1_RAT	0.579
**Purkinje cell protein 4**	PCP4_RAT	0.599
**Nucleobindin-2**	NUCB2_RAT	1.664
**Nucleosome assembly protein 1-like 4**	NP1L4_RAT	1.667
**Transmembrane and coiled-coil domains protein 1**	TMCO1_RAT	1.739
**ADP-ribosylation factor 4**	ARF4_RAT	1.756
**Histone H2A type 1**	H2A1C_RAT	1.761
**Omega-amidase NIT2**	NIT2_RAT	1.762
**Prostaglandin reductase 1**	PTGR1_RAT	1.825
**Taste receptor type 2 member 124**	TR124_RAT	1.854
**Leukotriene A-4 hydrolase**	LKHA4_RAT	1.867
**cAMP-dependent protein kinase type I-alpha regulatory subunit**	KAP0_RAT	1.885
**ADP-ribosylation factor 5**	ARF5_RAT	1.916
**Importin subunit alpha-5**	IMA5_RAT	1.931
**WD40 repeat-containing protein SMU1**	SMU1_RAT	1.965
**Large neutral amino acids transporter small subunit 1**	LAP2_RAT	1.988
**GPI transamidase component PIG-S**	PIGS_RAT	2.016
**Protein transport protein Sec31A**	SC31A_RAT	2.018
**Beta-crystallin S**	CRBS_RAT	2.096
**Ornithine aminotransferase, mitochondrial**	OAT_RAT	2.107
**Interphotoreceptor matrix proteoglycan 2**	IMPG2_RAT	2.132
**Gamma-crystallin F**	CRGF_RAT	2.215
**Nucleosome assembly protein 1-like 1**	NP1L1_RAT	2.286
**Major urinary protein**	MUP_RAT	2.401
**MARCKS-related protein**	MRP_RAT	2.410
**Flotillin-2**	FLOT2_RAT	2.522
**D-3-phosphoglycerate dehydrogenase**	SERA_RAT	2.625
**Monocarboxylate transporter 1**	MOT1_RAT	2.981
**Chloride intracellular channel protein 1**	CLIC1_RAT	3.165

### Western Blots Validating the MS Data

**Figure 3:**
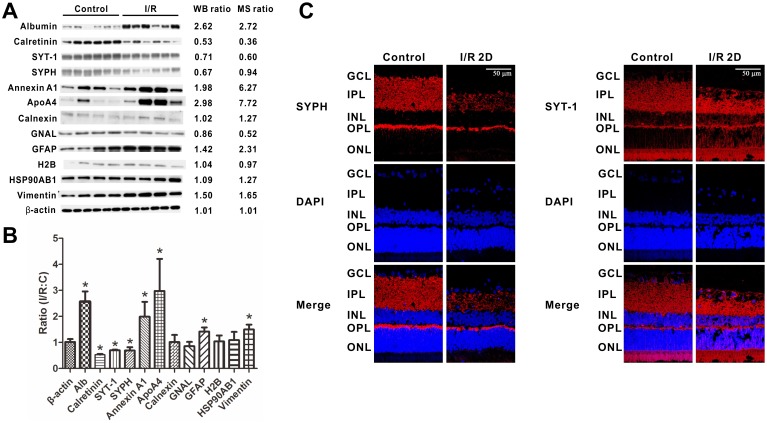
Western blot validation and IHC analysis of synaptophysin and synaptotagmin-1. The representative western blots of β-actin, albumin, calretinin, SYPH, SYT-1, Annexin A1, ApoA4, calnexin, GNAL, GFAP, H2B, HSP90AB1 and vimentin shown as the expression levels in the retinas (A) and as the densitometric quantitative results (B). Equal amounts of protein from the control retinas and the I/R-treated retinas were loaded. Each lane represents individual retina (n = 4 or 6 in each group; *p<0.05 compared with the non-injured retinas). The error bars represent the standard error of the mean. C, Retinal I/R-induced reductions of the synaptic proteins SYPH and SYT-1. The representative western blots of synaptophysin (SYPH) and synaptotagmin-1 (SYT-1) shown as the expression levels in the retinas are seen in (A). The stained retinal sections of SYPH (B) and SYT-1 (C) are shown at 2 days after the injury. Blue color: DAPI stained nuclei as control. Red color: SYPH or SYT-1 positively stained synapses. The scale bar represents 50 µm. C, non-injured eyes; I/R, I/R-injured eyes.

The quantitative proteomics data were obtained using mixed retinas. To provide additional evidence supporting the MS-based protein quantitation results, western blots were performed on selected proteins. For each retina from an individual adult rat, equal amounts (5 µg) of total RIPA lysate were loaded, and β-actin was used as a loading control. As shown in Fig. 3A and B: Calretinin, synaptotagmin-1 (SYT-1) and synaptophysin (SYPH) were down-regulated while albumin, Annexin A1, ApoA4, GFAP and vimentin were up-regulated following the I/R injury, when compared to the control group, with p<0.05. Besides, the western blot results of calnexin, GNAL, H2B and HSP90AB1 didn’t exhibit significant changes upon the I/R injury. The up- or down-regulation and also the unchanges detected by the western blot is consistent with the MS results, thus verifying that our proteomics data were reliable.

### Retinal I/R-Induced Reductions of Synaptic Proteins

Because most of the down-regulated proteins in the STRING network analysis were synapse-related proteins, we were interested in this phenomenon and chose two synaptic proteins, synaptophysin (SYPH) and synaptotagmin-1 (SYT-1), for IHC analysis. SYPH and SYT-1 are both integral membrane proteins associated with synaptic vesicles at synaptic terminals and are involved in neurotransmitter uptake and release [27], [28]. The western blot results showed that these two proteins were decreased following the I/R injury (Fig. 3A). Using immunofluorescence staining, we analyzed the location of SYPH and SYT-1 on retinal sections. As shown in Fig. 3C, SYPH, which is expressed in the inner plexiform layer (IPL) and outer plexiform (OPL), was significantly reduced following the retinal I/R injury, along with a reduction in the thicknesses of the IPL and OPL. The density of the SYPH-positive stained synapses also decreased, especially in the IPL. Meanwhile, the expression level of SYT-1 decreased only following the reduction in retinal thicknesses after the I/R injury (Fig. 3C). For SYT-1 and SYPH, the IHC image showed a significant decline consistent with the WB result, which suggests that the synapse-related proteins are actually down-regulated upon retinal I/R injury.

### I/R Injury Induces Ribosome Accumulation While Inhibiting the mTOR Pathway

**Figure 4:**
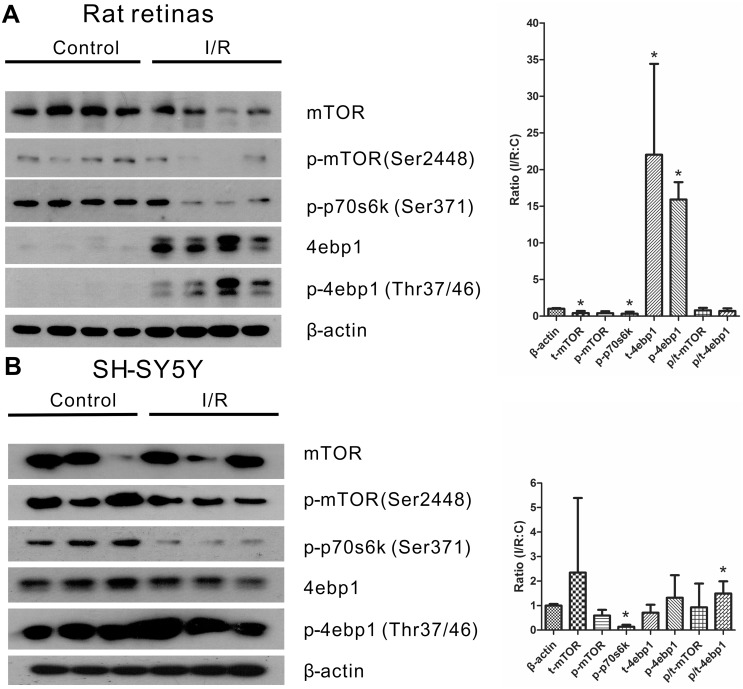
The mTOR pathway is suppressed upon I/R injury both *in vivo* and *in vitro*. Representative western blots and the densitometric quantitative results of mTOR, p-mTOR, p-p70 S6 kinase(p-p70s6k), 4E-BP1(4ebp1) and p-4E-BP1(p-4ebp1) showing the expression levels in the retinas (A) and in the SH-SY5Y cell line (B) upon I/R injury. A, Equal amounts of protein from the control retinas and the I/R-treated retinas were loaded. Each lane represents individual retina (n = 4 in each group; *p<0.05 compared with the non-injured retinas). B, Equal amounts of protein from the control and the I/R-treated SH-SY5Y cell were loaded. Each lane means individual biological replicates (n = 3 in each group; *p<0.05 compared with the non-injured retinas). The error bars represent the standard error of the mean.

In the STRING analysis, we observed a noticeably up-regulated protein network consisting of ribosomal proteins (Fig. 2). It is well known that ribosomal proteins play a crucial role in protein synthesis during translation. Numerous former studies have documented the mTOR pathway as an important pathway in the regulation of protein synthesis [29]. We further investigated any possible changes in the mTOR pathway following an I/R injury both *in vivo* and *in vitro*. Following the 2-day retinal I/R injury, the protein level of p-mTOR (Ser 2448) was down-regulated, and as a consequence, the downstream protein p-p70s6k (Ser 371) decreased as well, while the translation inhibitor 4ebp1 was up-regulated (Fig. 4A), which suggests that the protein synthesis mediated by the activation of mTOR is inhibited after retinal I/R injury. In addition, the activity of the mTOR pathway following *in vitro* I/R injury in SHSY5Y cells is consistently partially suppressed, with a significant down-regulation of p-p70s6k (Ser 371) only (Fig. 4B). These data provide evidence that the mTOR pathway is inhibited in the I/R model both *in vivo* and *in vitro*.

## Discussion

High intraocular pressure (HIOP) induced experimental retinal I/R has been used as a model to investigate the mechanism of retinal vascular- and neurodegeneration as well as the interaction of retinal cells [2]. In this model, neuron loss in the retinal ganglion cell layer occurred rapidly after injury, and vascular degeneration occurred subsequently [30]. In an earlier study, using a 0.05% curcumin diet as an intervention 2 days after the I/R injury, we found that the vascular degeneration can be inhibited, even following severe neuron loss, suggesting that the death signaling from dead neurons may not be effectively transferred to retinal vessels [20]. Therefore, the molecular changes during this time period may have an impact on retinal cell fate, determining cell survival or death after injury.

Quantitative proteomics analysis has emerged in recent years as an effective strategy to analyze protein changes [31]. In this study, we made a significant expansion (∼3-fold) on proteome coverage compared to previous proteomics studies ([15], [32]) on I/R injury, largely due to the use of stable isotope dimethyl labeling and extensive peptide fractionation. In total, we identified 1223 proteins and quantified 1088 proteins. Approximately 22% of the quantified proteins exhibited a greater than 1.5-fold change, including 40 down-regulated proteins and 194 up-regulated proteins. This shows that dramatic changes occur during I/R injury, and this phenomenon may be caused by the coordinated work of several pathways. Our proteomics data provide a global profile of protein changes upon I/R injury.

Gene Ontology classification and statistical enrichment revealed that one term in the biological process category, ‘metabolic process’, was overrepresented in the up-regulated proteins, but three other terms, ‘cell communication’, ‘system process’ and ‘transport’, were overrepresented in the down-regulated proteins (Fig. 1B). These processes give a global view of the cellular response, suggesting that following I/R injury, the retina exhibits a rise in basic metabolic activities and a decline in functional processes.

The STRING network analysis of the altered proteins provided further insights on the protein networks that may be involved in I/R injury. Two networks of proteins in the up-regulated proteins stand out: a ribosomal protein network and a secreted protein network (Fig. 2). The secreted proteins consist of many protease inhibitors. A previous study linked the calcium-activated protease calpain to retinal pathology during retinal ischemia [33]. Therefore, elevating the levels of certain protease inhibitors may be beneficial for recovery. The up-regulation of ribosomal proteins and protein synthesis-related accessory proteins (e.g., Eef2, Eef1d, Eef1g), as well as the elevation of protease inhibitors, suggest an elevated level of protein synthesis. However, the accumulation of ribosomal proteins as well as the inhibition of the mTOR pathway after I/R injury was observed in the same retinal I/R model. As contradictory as it may seem, these results are both supported by previous related studies. A mouse model overexpressing mutant human tau to mimic neurodegenerative diseases, such as Alzheimer’s disease, revealed that mass ribosomes (or polyribosomes) accumulate in degenerated neurons [34], [35]. In addition, previous studies have proven that the activity of the mTOR pathway is suppressed in axotomized retinal ganglion cells, which contributes to their failure to regenerate. Furthermore, activation of the mTOR pathway successfully leads to axon regeneration [36]. Additionally, the overexpression of cardiac mTOR protects against I/R injury-induced heart failure and sufficiently enhances both mortality and function [37]. Combined with our down-regulated mTOR pathway, the active mTOR pathway is essential for the injured cell to regenerate or survive in different disease models. It is possible that, after irreversible I/R injury, retinal neuronal and vascular cells tend to degenerate in addition to the inhibition of the mTOR pathway. Due to this inhibition, protein synthesis is shut down, and as a result, the ribosomal proteins facilitating protein translation accumulate abnormally in the injured cells. Further studies are required to elucidate the possible mechanisms.

Among the down-regulated protein networks, the synapse-related protein network stands out. The decreases of synapse proteins in I/R-injury retinas implies that synapse-related proteins may play an important role in retinal degeneration, predicting that there may be a functional reduction of retinal neurons in retinal ischemia. Immunohistochemistry studies on two synaptic proteins, synaptophysin (SYPH) and synaptotagmin-1 (SYT-1), validated this hypothesis. In the IHC results, both the SYPH expression and density were significantly decreased, while SYT-1 decreased only with the reduction of retinal thicknesses following the I/R injury (Fig. 3C). SYPH and SYT-1 play important roles in regulating exocytosis, neurotransmitter uptake, vesicle targeting and fusion with the presynaptic plasma membrane [27], [28]. This indicates that there is a functional reduction of retinal neurons upon I/R injury. Two recent studies, also using the HIOP model in rats, reported the expression and distribution of SYPH after injury [5], [38] and are consist with our findings of SYPH expression in the INL and OPL (Fig. 3C). Another study showed that synaptic plasticity can be induced by HIOP in retinas [5]. Therefore, the decrease of synapse-related proteins may also be connected to synaptic plasticity.

In Table 1, we listed 36 proteins, all with a greater than 1.5-fold change based on the MS analysis, that were not connected to other proteins in the STRING network analysis. These proteins included many calcium-binding and ion-transport proteins. Therefore, our proteomics analysis provides useful information for related mechanistic studies in retinal degeneration.

In summary, using stable isotope dimethyl labeling combined with SCX fractionation, we discovered the largest scale of proteome alteration upon retinal I/R injury to date. Through bioinformatics analyses and western blot, our study revealed a significant up-regulation of ribosomal proteins despite of the suppression of the mTOR pathway following an I/R injury. We also found a significant down-regulation of synapse-related proteins, which is most likely caused by the functional loss of retinal neurons. This provides new insights to elucidate the mechanism of neuronal degeneration in retinal I/R-injury research.

## Supporting Information

S1 Fig **The H & E stained retinal sections upon retinal I/R injury.** C: non-injured eyes; I/R 2D: I/R-injured eyes 2 days after the injury; I/R 5D: I/R-injured eyes 5 days after the injury. Size: 200 µm length per picture.
(TIF)

S2 Fig **Proteomics data overview of retinal I/R injury.** A, A Venn diagram of the proteins identified in the two replicates; B, A Venn diagram of the proteins quantified in the two replicates.
(TIF)

S3 Fig **The IHC analysis of albumin.** The stained retinal sections of albumin are shown at 2 days after the injury. Yellow color: albumin positively stained. C, non-injured eyes; I/R, I/R-injured eyes.
(TIF)

S1 Table **A summary of proteomics data.** Sheet 1, A list of all of the peptides identified in biological replicate 1; Sheet 2, A list of all of the peptides identified in biological replicate 2; Sheet 3, A list of all of the peptides quantified in biological replicate 1 (‘fraction’, ‘correlation’ and ‘standard error' were set at 0.5, 0.9 and 0.2); Sheet 4, A list of all of the peptides quantified in biological replicate 2 (‘fraction’, ‘correlation’ and ‘standard error’ were set at 0.5, 0.9 and 0.2); Sheet 5, A list of proteins quantified in both replicates with weight average ratio and SD values of two biological replicates.
(XLS)
