# Supplementary figures and images for: Identification of Protein Network Alterations upon Retinal Ischemia-Reperfusion Injury by Quantitative Proteomics Using a Rattus norvegicus Model
Source: PLoS One. 2014 Dec 30;9(12):e116453. doi: 10.1371/journal.pone.0116453 (PMC4280217; doi:10.1371/journal.pone.0116453)

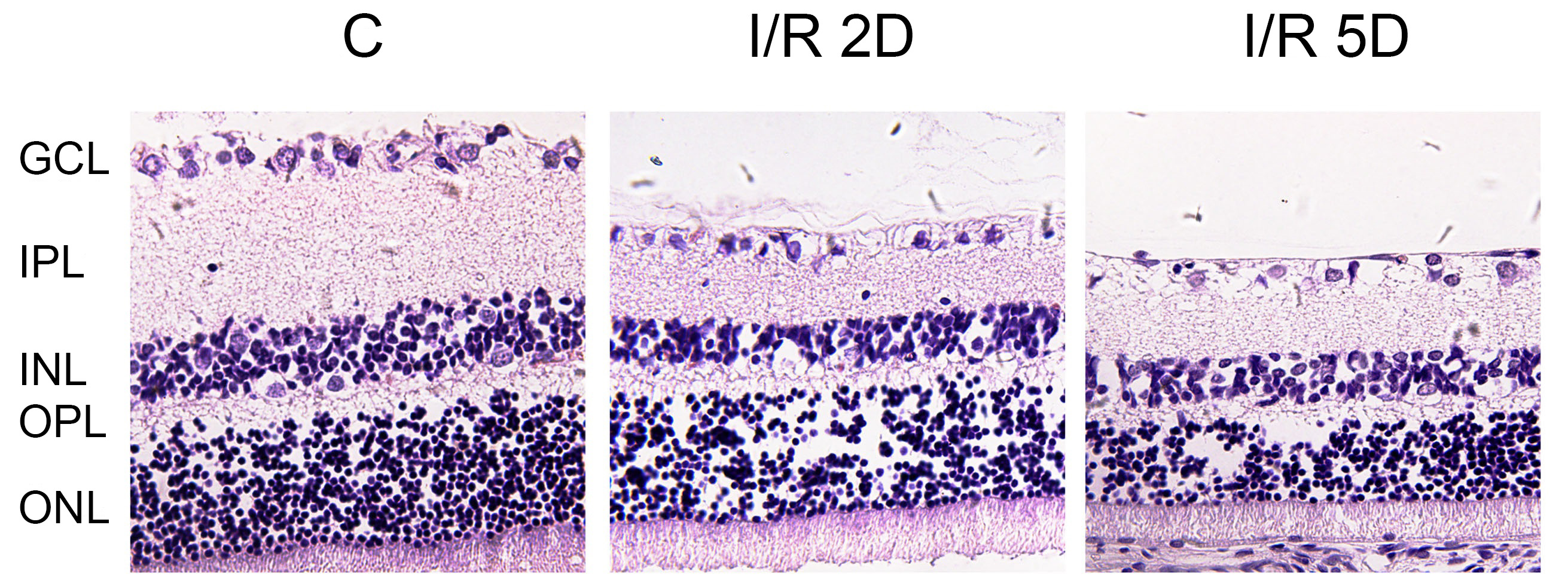

Supplement: S1 Fig — The H & E stained retinal sections upon retinal I/R injury. C: non-injured eyes; I/R 2D: I/R-injured eyes 2 days after the injury; I/R 5D: I/R-injured eyes 5 days after the injury. Size: 200 µm length per picture. (TIF) [file pone.0116453.s001.tif]

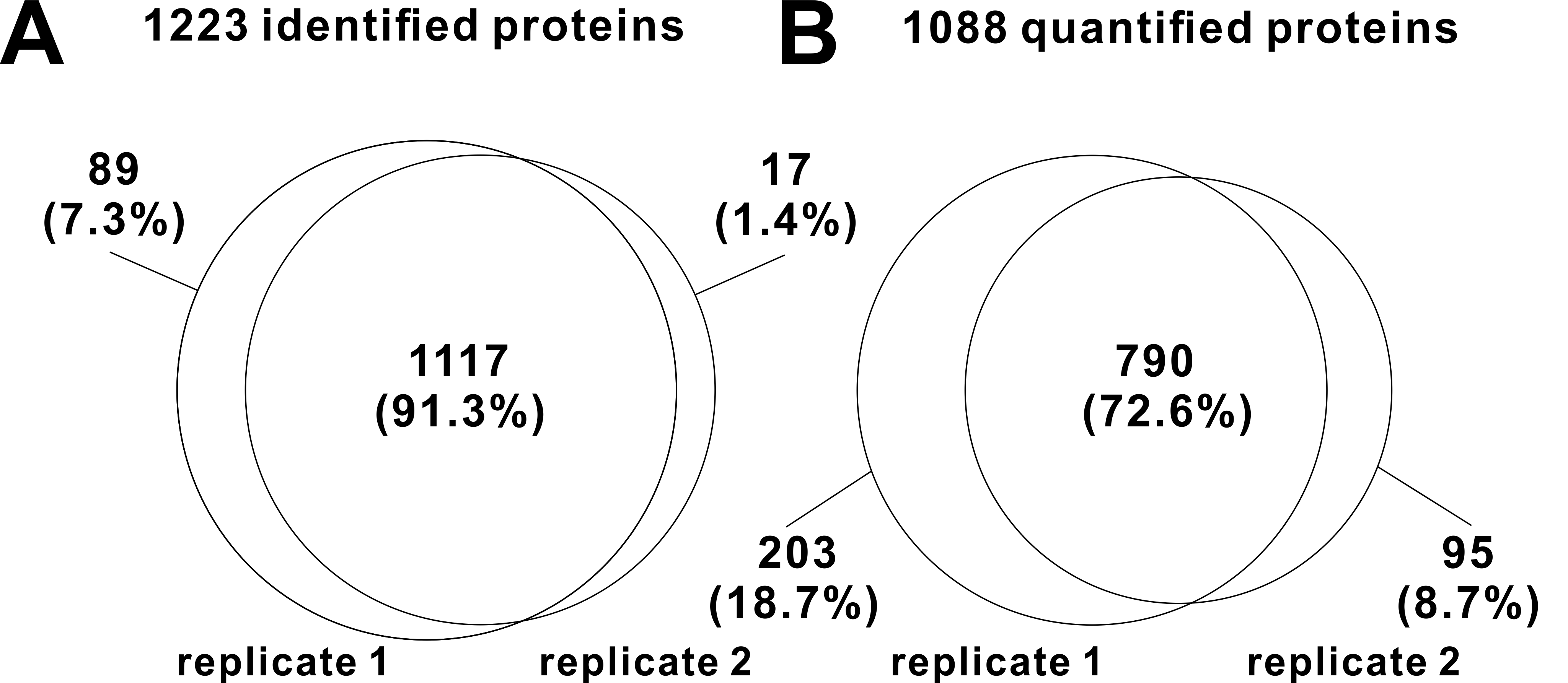

Supplement: S2 Fig — Proteomics data overview of retinal I/R injury. A, A Venn diagram of the proteins identified in the two replicates; B, A Venn diagram of the proteins quantified in the two replicates. (TIF) [file pone.0116453.s002.tif]

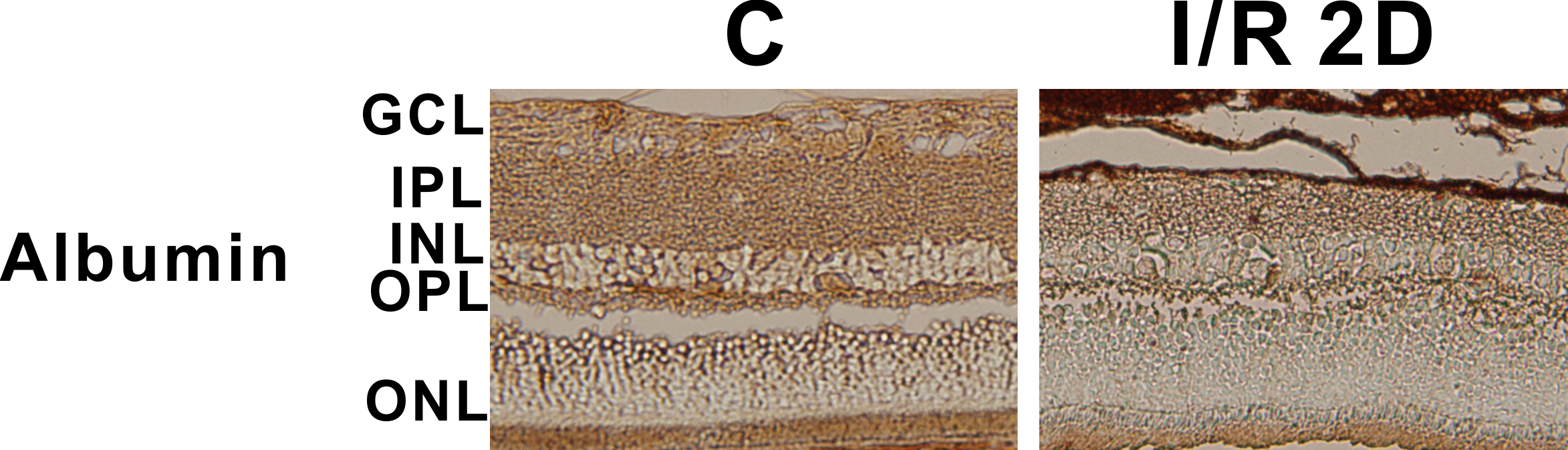

Supplement: S3 Fig — The IHC analysis of albumin. The stained retinal sections of albumin are shown at 2 days after the injury. Yellow color: albumin positively stained. C, non-injured eyes; I/R, I/R-injured eyes. (TIF) [file pone.0116453.s003.tif]
